# Outcome of elderly patients with diffuse large B-cell lymphoma treated with R-CHOP: results from the UK NCRI R-CHOP14v21 trial with combined analysis of molecular characteristics with the DSHNHL RICOVER-60 trial

**DOI:** 10.1093/annonc/mdx128

**Published:** 2017-04-07

**Authors:** A. Kühnl, D. Cunningham, N. Counsell, E. A. Hawkes, W. Qian, P. Smith, N. Chadwick, A. Lawrie, P. Mouncey, A. Jack, C. Pocock, K. M. Ardeshna, J. Radford, A. McMillan, J. Davies, D. Turner, A. Kruger, P. W. Johnson, J. Gambell, A. Rosenwald, G. Ott, H. Horn, M. Ziepert, M. Pfreundschuh, D. Linch

**Affiliations:** 1 Department of Medicine, The Royal Marsden NHS Foundation Trust, London and Surrey;; 2 Cancer Research UK and UCL Cancer Trials Centre, UCL Cancer Institute, London, UK;; 3 Olivia-Newton John Cancer Research & Wellness Centre, Melbourne, Australia;; 4 Department of Oncology, Cambridge University Hospitals NHS Foundation Trust, Cambridge;; 5 HMDS, St James’s Institute of Oncology, Leeds;; 6 East Kent Hospitals, Canterbury;; 7 Department of Hematology, University College London, London;; 8 Mount Vernon Cancer Centre, Northwood;; 9 Department of Medical Oncology, University of Manchester and the Christie NHS Foundation Trust, Manchester;; 10 Department of Hematology, Nottingham City Hospital, Nottingham;; 11 Western General Hospital, Edinburgh;; 12 Department of Hematology, Torbay Hospital, Torquay;; 13 Royal Cornwall Hospital, Truro;; 14 Cancer Research UK Center, University of Southampton, Southampton, UK;; 15 Institute of Pathology, Würzburg University, Würzburg;; 16 Department of Clinical Pathology, Robert-Bosch-Krankenhaus, Stuttgart;; 17 Dr. Margarete Fischer-Bosch Institute of Clinical Pharmacology, Stuttgart and University of Tübingen, Stuttgart;; 18 Institute for Medical Informatics, Statistics, and Epidemiology, University of Leipzig, Leipzig;; 19 Department of Medicine, Saarland University Medical School, Homburg/Saar, Germany

**Keywords:** diffuse large B-cell lymphoma, elderly, R-CHOP, MYC, double-hit lymphoma

## Abstract

**Background:**

There is an on-going debate whether 2- or 3-weekly administration of R-CHOP is the preferred first-line treatment for elderly patients with diffuse large B-cell lymphoma (DLBCL). The UK NCRI R-CHOP14v21 randomized phase 3 trial did not demonstrate a difference in outcomes between R-CHOP-14 and R-CHOP-21 in newly diagnosed DLBCL patients aged 19–88 years, but data on elderly patients have not been reported in detail so far. Here, we provide a subgroup analysis of patients ≥60 years treated on the R-CHOP14v21 trial with extended follow-up.

**Patients and methods:**

Six hundred and four R-CHOP14v21 patients ≥60 years were included in this subgroup analysis, with a median follow-up of 77.7 months. To assess the impact of *MYC* rearrangements (*MYC*-R) and double-hit-lymphoma (DHL) on outcome in elderly patients, we performed a joint analysis of cases with available molecular data from the R-CHOP14v21 (*N *=* *217) and RICOVER-60 (*N *=* *204) trials.

**Results:**

Elderly DLBCL patients received high dose intensities with median total doses of ≥98% for all agents. Toxicities were similar in both arms with the exception of more grade ≥3 neutropenia (*P *<* *0.0001) and fewer grade ≥3 thrombocytopenia (*P *=* *0.05) in R-CHOP-21 versus R-CHOP-14. The elderly patient population had a favorable 5-year overall survival (OS) of 69% (95% CI: 65–73). We did not identify any subgroup of patients that showed differential response to either regimen. In multivariable analysis including individual factors of the IPI, gender, bulk, B2M and albumin levels, only age and B2M were of independent prognostic significance for OS. Molecular analyses demonstrated a significant impact of *MYC*-R (HR = 1.96; 95% CI: 1.22–3.16; *P *=* *0.01) and DHL (HR = 2.21; 95% CI: 1.18–4.11; *P *=* *0.01) on OS in the combined trial cohorts, independent of other prognostic factors.

**Conclusions:**

Our data support equivalence of both R-CHOP application forms in elderly DLBCL patients. Elderly *MYC*-R and DHL patients have inferior prognosis and should be considered for alternative treatment approaches.

**Trial numbers:**

ISCRTN 16017947 (R-CHOP14v21); NCT00052936 (RICOVER-60).

## Introduction

Elderly patients with diffuse large B-cell lymphoma (DLBCL) have a worse prognosis compared to the younger patient population. This is partly explained by lower treatment tolerability in elderly patients with difficulties to administer adequate doses of chemotherapy. However, even when receiving comparable treatment intensities, elderly DLBCL patients have inferior outcome, potentially indicating more aggressive disease biology. Therefore, dose-intensified administration of R-CHOP immunochemotherapy might be of particular benefit for elderly DLBCL patients to overcome these high-risk factors. Treatment of patients >60 years (y) with 6× R-CHOP-14 plus 2× rituximab in the German RICOVER-60 trial has achieved the best long-term outcome in elderly DLBCL patients published to date [1]. However, superiority of dose-intensified R-CHOP-14 compared to the 3-weekly administration in elderly DLBCL patients could not be demonstrated in randomized trials.

The GELA LNH03-6B trial comparing R-CHOP-14 and R-CHOP-21 in DLBCL patients aged 60–80y showed no difference of either regimen [2], but results were criticized due to high treatment-related mortality and low dose intensities in the R-CHOP-14 arm. The UK NCRI R-CHOP14v21 trial compared the 2- and 3-weekly R-CHOP regimens in DLBCL patients aged 18–88y and similarly did not observe a difference in outcome across age groups [3]. However, outcomes of the elderly R-CHOP14v21 trial cohort have not been reported separately and it remained unclear whether particular subgroups of elderly patients benefit from intensified treatment.

The International Prognostic Index (IPI) is widely used for prognostication of younger and elderly DLBCL patients. Due to differences in disease biology and outcomes it has been proposed to use separate prognostic scores for the elderly patient group [4, 5], but these have not yet been validated in large independent cohorts.

Several molecular high-risk markers have been identified in DLBCL that could potentially refine clinical prognostic models. Cell-of-origin (COO) assessment of DLBCL according to gene-expression-profiling separates the germinal center B-cell (GCB) and the poor prognostic activated B-cell (ABC) subtypes, but these analyses lack prospective validation and methodological problems currently limit their use in standard practice. The negative prognostic impact of *MYC* rearrangements (*MYC*-R) as well as *MYC*- and concomitant *BCL2*- or *BCL6* rearrangements (double-hit lymphoma; DHL) has been shown in several DLBCL cohorts [6, 7]. The prognostic significance of *MYC*-R seems to be particularly high in older DLBCL patients [6]. Due to the low incidence of *MYC*-R and DHL and possibly due to their age-dependent relevance, an independent prognostic significance of these markers in multivariate models has not yet been demonstrated in prospective trial cohorts of R-CHOP-treated patients.

The aim of this subgroup analysis was to provide detailed outcomes and toxicity data on elderly patients treated within the R-CHOP14v21 trial and to investigate the impact of clinical and molecular factors on outcome in this age group.

## Patients and methods

Patient characteristics in the R-CHOP14v21 trial have been published in detail [3]. A brief description of the trial is given in the Supplement (available at *Annals of Oncology* online).

Of 1080 R-CHOP14v21 patients, 604 were ≥60y and included in the current analysis. Details of statistical analyses are provided in the Supplement.

COO was assessed by the immunohistochemistry (IHC)-based Hans algorithm. Assessment of *MYC*-, *BCL2*- and *BCL6-*rearrangements was done with fluorescence *insitu* hybridization (FISH; *N *=* *217). DHL was defined as presence of *MYC*- and either *BCL2-* or *BCL6-*rearrangements. In order to increase the sample size to assess the impact of *MYC*-R and DHL on outcome in elderly DLBCL patients, we performed a joint analysis with data from 204 elderly DLBCL patients treated on the RICOVER-60 trial who had molecular results available (supplementary Table S1, available at *Annals of Oncology* online). Details of the German high*-*grade non-Hodgkin lymphoma study group (DSHNHL) RICOVER-60 trial and methods of molecular analyses within the trial have been previously described [1, 7].

## Results

We included 604 elderly patients from the R-CHOP14v21 trial in this subgroup analysis. Patients’ median age was 67y (range 60–88). Baseline characteristics were well balanced between treatment arms (Table 1). There was a trend towards a higher rate of *BCL6* rearrangements and DHL in R-CHOP-14 (*P *=* *0.10 and *P *=* *0.06, respectively).

**Table 1:** Baseline characteristics

Characteristics	R-CHOP-21	R-CHOP-14
	(*N*=301)	(*N*=303)
	*n* (%)	*n* (%)
Age (years)		
60–69	192 (64)	196 (65)
≥70	109 (36)	107 (35)
Sex		
Female	148 (49)	150 (50)
Male	153 (51)	153 (50)
WHO performance status		
0	120 (40)	143 (47)
1	132 (44)	118 (39)
2	49 (16)	42 (14)
Stage (*N*=596)		
IA	9 (3)	9 (3)
IB	6 (2)	7 (2)
II	90 (30)	83 (28)
III	91 (31)	104 (35)
IV	102 (34)	95 (32)
Bulk (*N*=601)	139 (47)	126 (42)
B symptoms	121 (40)	134 (44)
Elevated LDH	200 (66)	197 (65)
>1 extranodal sites	94 (31)	82 (27)
IPI score		
1	48 (16)	44 (15)
2	75 (25)	90 (30)
3	98 (33)	104 (34)
4	66 (22)	56 (18)
5	14 (5)	9 (3)
Subtype (*N*=317)		
GCB	76 (50)	82 (50)
Non-GCB	77 (50)	82 (50)
β2-microglobulin ≥3mg/L (*N*=371)	88 (51)	102 (52)
Albumin ≤35g/L (*N*=598)	100 (34)	86 (29)
*MYC* rearrangement (*N*=217)	9 (9)	14 (12)
*BCL2* translocation (*N*=220)	26 (25)	33 (28)
*BCL6* rearrangement (*N*=218)	17 (16)	30 (26)
Double-hit abnormality (*N*=215)	5 (5)	9 (8)

Dose intensities were high in both trial arms. Median total doses of cyclosphosphamide, doxorubicin, vincristine, prednisolone and rituximab received were 98% versus 99%, 98% versus 99%, 100% versus 100%, 98% versus 100%, and 98% versus 98% in R-CHOP-21 and R-CHOP-14, respectively. Seventy-one (24%) patients on R-CHOP-21 and 46 (15%) patients on R-CHOP-14 did not complete all treatment cycles (*P *=* *0.01). Reasons for early treatment termination are listed in supplementary Table S2, available at *Annals of Oncology* online, with treatment-related toxicity being the most common cause. Frequency of dose reductions was similar in both arms (15% for R-CHOP-21 versus 16% for R-CHOP-14; *P *=* *0.73).

Treatment toxicities are given in Table 2. There was evidence of more grade ≥3 neutropenia (62% versus 36%; *P *<* *0.0001) and less grade ≥3 thrombocytopenia (7% versus 12%; *P *=* *0.05) in R-CHOP-21 compared to R-CHOP-14. Patients on R-CHOP-21 had lower incidence of anemia (20% versus 31%; *P *=* *0.001), with a similar trend for grade ≥3 anemia (2% versus 5%; *P *=* *0.11). No significant difference in the incidence of fever and infections or any other toxicity was observed. The incidence of treatment-related deaths, fatal cardiac events and secondary malignancies were similar in both arms (supplementary Table S3, available at *Annals of Oncology* online).

**Table 2:** Most common grade ≥3 toxicities and cause of treatment-related deaths

	R-CHOP-21 (*N*=301)		R-CHOP-14 (*N*=303)	
	Any grade	Grade ≥3	Any grade	Grade ≥3
All toxicities	292 (97%)	216 (72%)	299 (99%)	182 (60%)
Neutropenia	224 (74%)	185 (61%)	138 (46%)	109 (36%)
Thrombocytopenia	73 (24%)	22 (7%)	112 (37%)	37 (12%)
Anemia	60 (20%)	6 (2%)	95 (31%)	14 (5%)
Infection	145 (48%)	71 (24%)	146 (48%)	71 (23%)
Fever	70 (23%)	16 (5%)	56 (18%)	16 (5%)
Mucositis	143 (48%)	4 (1%)	167 (55%)	8 (3%)
Nausea	188 (62%)	7 (2%)	151 (50%)	12 (4%)
Vomiting	98 (33%)	7 (2%)	82 (27%)	9 (3%)
Diarrhoea	109 (36%)	12 (4%)	113 (37%)	16 (5%)
Constipation	185 (61%)	7 (2%)	160 (53%)	8 (3%)
Neurological	167 (55%)	23 (8%)	183 (60%)	36 (12%)
Fatigue	240 (80%)	31 (10%)	252 (83%)	40 (13%)
Bone pain	68 (23%)	7 (2%)	102 (34%)	6 (2%)
Cardiac	29 (10%)	2 (1%)	29 (10%)	9 (3%)

Treatment-related deaths: 3 in R-CHOP-21: 2 nonneutropenic sepsis and 1 neutropenic sepsis; and 7 in R-CHOP-14: 2 nonneutropenic sepsis, 1 neutropenic sepsis, 1 renal failure and 3 not specified.

Response was assessable in 274 patients in each arm. There was no evidence of a difference in response rates between R-CHOP-21 and R-CHOP-14 [complete response (CR)/unconfirmed CR (CRu): 67% versus 62%, *P *=* *0.21; overall response rate (ORR) both 91%; Table 3]. CR/CRu rates after four cycles of therapy were 39% and 33%, respectively (*P *=* *0.15). 61% and 60% of patients are still alive without progression (supplementary Table S3, available at *Annals of Oncology* online). Four patients on R-CHOP-21 and seven on R-CHOP-14 presented with central nervous system relapse (*P *=* *0.55).

**Table 3:** Response to treatment

End of treatment response	R-CHOP-21	R-CHOP-14
	(*N*=274)	(*N*=274)
	*n* (%)	*n* (%)
Complete response (CR)	145 (53)	119 (43)
Unconfirmed complete response (CRu)	39 (14)	50 (18)
Partial response	64 (23)	80 (29)
Stable disease	16 (6)	16 (6)
Progressive disease or relapse	10 (4)	9 (3)
CR/Cru	184 (67)	169 (62)
Overall response rate	248 (91)	249 (91)

**Figure 1.**
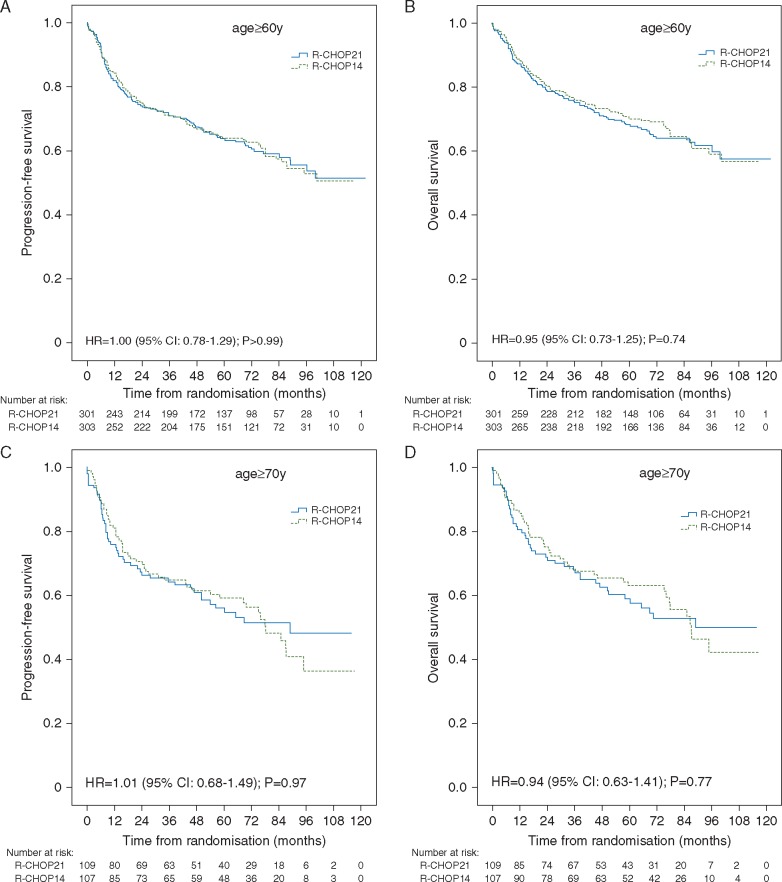
Kaplan–Meier curves of PFS and OS in (A-B) patients over 60 years and (C-D) patients over 70 years.

After a median follow-up of 77.7 months, there was no evidence of a difference in progression-free survival (PFS) and overall survival (OS) between treatment arms in patients ≥60y or ≥70y (Figure 1A-D). No difference in survival between R-CHOP-21 and R-CHOP-14 was observed in patients who only achieved partial response (PR) after four cycles (*P *=* *0.79 for PFS; *P *=* *0.68 for OS). There was also no difference between treatment arms with respect to gender (*P *=* *0.54 for PFS; *P *=* *0.67 for OS) or IPI (*P *=* *0.64 for PFS; *P *=* *0.50 for OS). 5y-PFS was 64% (95% CI: 60-68) in patients ≥60y and 58% (95% CI: 51-65) in patients ≥70y. 5y-OS was 69% (95% CI: 66–73) and 61% (95% CI: 54–68), respectively.

63/280 (23%) patients with available data received consolidation radiotherapy. Of those, 36 had initial bulk, 20 extranodal disease, and 10 had both. Disease status before radiotherapy was available for 61 patients: 23 (37%) CR/CRu, 31 (51%) PR and 7 (12%) SD. In patients with PR or SD who are supposed to benefit most from radiotherapy, the use of radiotherapy was not associated with OS (supplementary Figure S3, available at *Annals of Oncology* online).

In multivariable analysis, only age and B2M levels were of independent prognostic significance for OS (supplementary Table S4, available at *Annals of Oncology* online). There was no significant impact of COO subtypes on outcomes (supplementary Figure S1, available at *Annals of Oncology* online). When comparing prognostic scores IPI, R-IPI, E-IPI and ABE4 (supplementary Table S5 and Figure S2, available at *Annals of Oncology* online), ABE4 achieved the best fit and discrimination for predicting OS, followed by the IPI. Similar results were obtained for PFS (data not shown).

**Figure 2.**
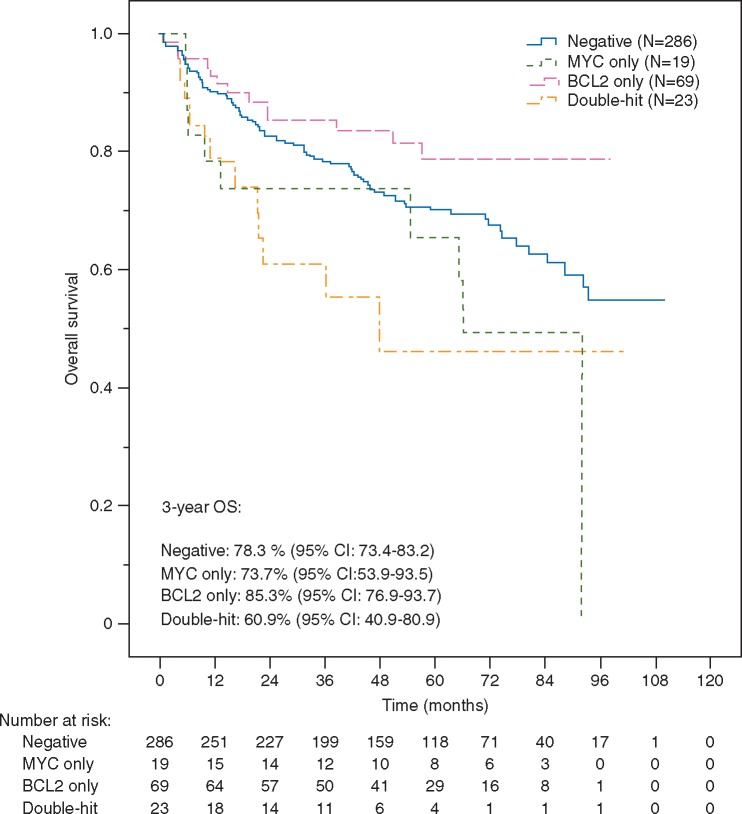
Kaplan–Meier curves of OS according to *MYC-* and *BCL2* rearrangements and double-hit abnormality in R-CHOP treated elderly patients from R-CHOP14v21 (*N *=* *215) and RICOVER-60 (*N *=* *182).

To assess the impact of *MYC*-R and DHL on outcome we performed a joint analysis with cases from RICOVER-60. 23/217 (11%) patients from our cohort and 19/204 (9%) patients from RICOVER-60 had *MYC*-R as determined by FISH. 14/215 (7%) and 9/182 (5%) had DHL, respectively. *MYC*-R and DHL cases had significantly worse OS compared to cases without these abnormalities [HR = 1.96 (95% CI: 1.22–3.16); *P *=* *0.01 and HR = 2.21 (95% CI: 1.18–4.11); *P *=* *0.01, respectively); Figure 2]. Similar effect sizes were observed after adjusting for individual IPI factors and trial arms [HR = 1.76 (95% CI: 1.09–2.85); *P *=* *0.02 and HR = 2.08 (95% CI: 1.11–3.90); *P *=* *0.02, respectively)]. The difference in OS between DHL and *MYC*-R was not significant (HR = 1.38 (95% CI: 0.55–3.43; *P *=* *0.49). There was no significant impact of *BCL2*- or *BCL6*-rearrangements on OS (*P *=* *0.34 and *P *=* *0.99, respectively).

## Discussion

With a median follow-up of 6.5y, we provide a detailed analysis of outcome and toxicities from patients with newly diagnosed DLBCL aged ≥60y treated on the phase 3 R-CHOP14v21 trial.

Elderly DLBCL patients in our cohort had an excellent long-term outcome with 5y-OS of 69% (3y-PFS 71%; 3y-OS 76%). These results are similar to data from elderly DLBCL patients treated with 6× R-CHOP-14 on RICOVER-60 (3y-PFS 73%; 3y-OS 78%) and better than outcomes in the GELA LNH03-6B trial (3y-PFS 61%; 3y-OS 73%) [1, 2]. Of note, patients’ median age was higher in LNH03-6B (70y) compared to our cohort (67y) and RICOVER-60 (68y). In addition, there were more cases presenting with high IPI (3–5) in the LNH03-6B trial (75% versus 57% in our subgroup versus 43% in RICOVER-60), which might have contributed to inferior outcome seen in this trial population.

Toxicity profiles in our cohort of elderly DLBCL patients were favorable in both treatment arms. As expected, patients on R-CHOP-21 had a higher incidence of neutropenia probably due to reduced use of G-CSF, but less thrombocytopenia. Importantly, there was no difference in infectious complications or treatment-related deaths. The incidence of deaths during chemotherapy was very low at 1.7%, suggesting adequate management of elderly patients in participating centers. In the LNH03-6B trial, a high treatment-related mortality of 9% was observed in the initial recruitment period, which improved towards the end of the study, indicating gain of clinical experience with dose-intensified treatment in elderly patients. With 6.5y median follow-up, there was no difference in long-term toxicity, specifically cardiac events and secondary malignancies, between arms.

Dose intensities were high in both arms and as seen in the entire R-CHOP14v21 trial cohort [3]. The low dose intensity of 88% for R-CHOP-14 in the LNH03-6B trial could have potentially underestimated efficacy of the 2-weekly regimen. Our results support equivalence of both regimens in elderly DLBCL patients when adequate doses are achieved. However, the study was not powered for this *posthoc* subgroup analysis in elderly patients.

We did not identify any subgroup of elderly DLBCL patients that showed differential response to either regimen, including gender and IPI groups. No difference between treatment arms could be seen in patients ≥70y. An analysis of patients ≥80y was not feasible due to low numbers (*N *=* *20). Moreover, there was no benefit of dose-intense treatment in late responders who had not achieved CR/Cru after four cycles.

Consolidation radiotherapy was at the discretion of the investigators and performed in 23% of elderly patients with available data. The main indication for radiotherapy was initial bulky or extranodal disease. The benefit of radiotherapy to initial bulk in elderly DLBCL patients is reported to be greatest for patients who are not in CR/CRu after induction therapy [8]. Accordingly, most patients in our analysis received radiotherapy to PR or SD at the end of treatment, without evidence of a survival benefit for this strategy. However, these data have significant limitations (nonrandomized approach, small numbers). In addition, no PET-CT data were recorded. The on-going DSHNHL OPTIMAL > 60 trial will investigate whether consolidation radiotherapy can be safely omitted in elderly DLBCL patients who are PET-negative at the end of treatment.

Remarkably, PFS of elderly patients was only 8 percentage points worse at 5y compared to younger patients (5y-PFS 64% versus 72%), supporting the concept of treating elderly patients with full doses of chemotherapy whenever possible. Toxicities were also similar between elderly and younger patients (data not shown), besides a significantly higher rate of grade ≥3 neutropenia in elderly (*P*≤0.001).

Differences between DLBCL of elderly and younger patients have been described on the molecular level, with higher frequencies of ABC subtypes, *BCL6* rearrangements, gains in 1q21, 18q21, and 7q21, and a higher genetic complexity associated with increasing age [9]. We did not observe material differences in the frequency of *MYC*-R, *BCL6*- and *BCL2*-rearrangements between age groups, nor in the incidence of IHC-based cell-of-origin subtypes (data not shown). We found lower frequency of bulky disease and higher B2M levels in elderly compared to younger patients, implying differences in disease biology between both groups.

Age-specific clinical and molecular features suggest the need for a separate prognostic scoring system for elderly patients. We compared performances of two recently proposed prognostic scores for elderly DLBCL (ABE4 [5] and E-IPI [4]) with the standard IPI and R-IPI in our cohort. Both scores use an age cut-off of 70y. ABE4 further incorporates bulky disease and separates PS ≥ 1 instead of ≥ 2. The ABE4 performed best in our cohort, despite bulky disease not being significantly associated with patient outcomes. Therefore, separating patients with PS 0 from those with PS ≥ 1 could be a more appropriate cut-off in an elderly patient group. Both ABE4 and IPI distinguished meaningful prognostic groups for PFS and OS. However, clinical utility of the ABE4 score might be limited by the fact that only 9% of patients from our cohort were in the high-risk group compared with 14% in the original Czech Lymphoma Registry [5]. As discussed by Ziepert et al. [10], introduction of new scores have to be seen with caution and should only be considered if properly validated and if changing patients’ management. The main use of the IPI has been in the context of clinical trials, allowing risk-stratification of patients and facilitating comparison of results across trials. A NCCN-IPI has recently been proposed which separates three different age groups as risk factors [11]. The great disadvantage of this score is that it cannot be used for elderly and young patient groups separately and is therefore unsuitable for age-specific DLBCL trials. In contrast, the IPI as age-adjusted IPI has been validated in both young and elderly DLBCL.

In line with previous findings, IHC-based cell-of-origin classification did not impact on outcomes, further underscoring limitations of this method. However, final analyses of the REMoDL-B trial will reveal if the concept of cell-of-origin classification as prognostic marker holds true when assessed prospectively [12]. Our combined analysis of FISH data from R-CHOP14v21 and RICOVER-60 demonstrates for the first time independent prognostic significance of both *MYC*-R and DHL in patients treated with R-CHOP within prospective cohorts. A negative prognostic impact of *MYC*-R and DHL has been reported in several heterogeneous DLBCL populations, but did not reach independent significance in trial cohorts due to small numbers [3, 7]. On-going prospective trials will reveal if these patients benefit from upfront treatment intensification.

In conclusion, our data demonstrate excellent short and long-term results with both R-CHOP-14 and R-CHOP-21 in elderly DLBCL patients. This analysis contributes important information to the longstanding discussion about optimal management of the elderly DLBCL patient population and provides a detailed analysis of molecular and clinical prognostic factors in this age group.

## Supplementary Material

Supplementary Data
