# Supplementary material for: Outcome of elderly patients with diffuse large B-cell lymphoma treated with R-CHOP: results from the UK NCRI R-CHOP14v21 trial with combined analysis of molecular characteristics with the DSHNHL RICOVER-60 trial
Source: Ann Oncol. 2017 Apr 7;28(7):1540–6. doi: 10.1093/annonc/mdx128 (PMC5815562; doi:10.1093/annonc/mdx128)
Supplement: Supplementary Data [file mdx128_supp.zip › mdx128-suppl_data/Supplement revision.docx]

**Supplement**

**Patients and Methods**

In the phase 3 UK NCRI R-CHOP14v21 trial, patients aged 18 years or older with untreated stage IA bulky or stage IB-IV DLBCL were randomly assigned between 2005 and 2008 to receive either 8 cycles R-CHOP-21 or 6 cycles R-CHOP-14 (+ G-CSF) with two additional rituximab applications. Doses of rituximab, cyclophosphamide, doxorubicin, vincristine and prednisolone were based on the original GELA and DSHNHL regimens. G-CSF was administered on days 4-12 of each cycle. 57% of patients in the R-CHOP-21 arm received G-CSF as secondary prophylaxis at the discretion of the investigators. All patients received antibiotic prophylaxis with co-trimoxazole until 2 weeks after the end of treatment. Performance of consolidation radiotherapy was at the discretion of the investigators. Response assessment was performed using contrast-enhanced CT scans after 4 cycles of therapy, at the end of treatment, and 3 months and 12 months after completion of treatment. Histological diagnosis was confirmed in central histopathology review including immunohistochemistry (IHC)-based cell-of-origin classification using the Hans algorithm. The R-CHOP14v21 trial was conducted according to the Declaration of Helsinki and all participants provided written informed consent.

604 patients in the R-CHOP14v21 trial were ≥60 years and included in the current subgroup analysis (301 in the R-CHOP-21 arm, 303 in the R-CHOP-14 arm). The median follow-up was 77.7 months.

For the joint analysis of *MYC*-R and DHL in elderly DLBCL patients, molecular data was available for 215 R-CHOP14v21 cases and 182 R-CHOP-treated patients from RICOVER-60. Patients in the combined cohorts with available molecular data were more frequently female (52% vs. 46%; *P*=0.05), had fewer WHO performance status 2 (11% vs. 17%; *P*=0.01), fewer bulky disease (33% vs. 45%; *P*<0.01) and fewer B symptoms (32% vs. 40%; *P*=0.01) compared to patients without molecular data. Molecular results were not blinded for outcome analyses.

## Statistics

The primary endpoint of the R-CHOP14v21 trial was overall survival (OS). Secondary outcome measures were response rate, progression-free survival (PFS) and toxicity.

Response was assessed by the local treating physician and categorised into complete response (CR), unconfirmed CR (CRu), partial response (PR), stable disease (SD) and progressive disease (PD) in accordance with the International Workshop Standardized Response Criteria for Non-Hodgkin Lymphoma. Adverse events were classified using the National Cancer Institute Common Terminology Criteria for Adverse Events (NCI-CTCAE, version 3.0). Patients who received at least one cycle of therapy were included in the toxicity analyses.

OS and PFS were calculated by the Kaplan-Meier method with the log-rank comparing differences between survival curves. Clinical findings were compared between groups using the χ2 or Mann-Whitney U test. A *P* value of ≤ 0.05 (two-sided) was regarded as significant. Multivariate analyses were performed using Cox logistic regression including the following variables: age as continuous variable, WHO performance status (PS) 0/1 vs. ≥2, LDH normal vs. >upper limit of normal (ULN), Stage I/II vs. III/IV, number of extranodal sites involved 0-1 vs. ≥2, gender, bulky disease (diameter ˃10cm) absent vs. present, β2-microglobulin (B2M) <3mg/L vs. ≥3mg/L, and albumin >35 g/L vs. ≤35 g/L.

The age-specific prognostic scores elderly IPI (E-IPI) and ABE4 were compared with standard IPI and revised IPI (R-IPI). In contrast to the original publication, bulky disease was defined as tumour of greater than 10cm (not 7.5cm) to calculate the ABE4 score. Performance of scores were compared by global fit criterion AIC (Akaike’s information criteria) and discrimination according to CPE (concordance probability estimate), with lower values of AIC indicating better fit and higher values of CPE better discrimination

**Supplementary Tables and Figures**

**Table S1: Clinical characteristics according to MYC-R and DHL**

| **Characteristics** | **non-MYC**  **(*N*=379)**  *n* (%) | **MYC-R**  **(*N*=42)**  *n* (%) | **non-DHL**  **(*N*=374)**  *n* (%) | **DHL**  **(*N*=23)**  *n* (%) |
| --- | --- | --- | --- | --- |
| Age (years)  60-69  ≥70 | 233 (61%)  146 (39%) | 25 (60%)  17 (40%) | 230 (61%)  144 (39%) | 14 (61%)  9 (39%) |
| Sex  Female  Male | 197 (52%)  182 (48%) | 18 (43%)  24 (57%) | 196 (52%)  178 (48%) | 11 (48%)  12 (52%) |
| Stage III/IV | 203 (54%) | 19 (45%) | 201 (54%) | 13 (57%) |
| WHO performance status >1 | 40 (11%) | 7 (17%) | 39 (10%) | 4 (17%) |
| Elevated LDH | 201 (53%) | 26 (62%) | 205 (55%) | 15 (65%) |
| >1 extranodal sites | 83 (22%) | 9 (21%) | 80 (21%) | 7 (30%) |
| IPI score  1  2  3  4  5 | 110 (29%)  93 (25%)  110 (29%)  57 (15%)  9 (2%) | 13 (31%)  10 (24%)  10 (24%)  6 (14%)  3 (7%) | 105 (28%)  95 (25%)  110 (29%)  54 (14%)  10 (3%) | 5 (22%)  5 (22%)  7 (30%)  4 (17%)  2 (9%) |
| Bulk | 118 (31%) | 19 (45%) | 121 (32%) | 11 (48%) |
| B symptoms | 120 (32%) | 17 (40%) | 118 (32%) | 9 (39%) |

**Table S2: Reasons for early termination of treatment**

| **Reason for early termination** | **R-CHOP-21**  **(*N*=301)**  ***n*** | **R-CHOP-14**  **(*N*=303)**  ***n*** |
| --- | --- | --- |
| Disease progression (PD)  -Death due to PD | 7  1 | 4  2 |
| Clinical decision | 8 | 1 |
| Patient refusal | 6 | 5 |
| Other medical condition  -Death due to other medical condition | 10  3 | 9  3 |
| Treatment-related toxicity  -Death related to treatment | 27  3 | 19  7 |
| Death, other cause or unknown  Diagnosis changed  Other | 2  3  7 | 1  4  2 |
| Not known/missing | 1 | 1 |

**Table S3: Survival status and cause of death**

| **Status and cause of death** | **R-CHOP-21**  **(*N*=301)**  ***n* (%)** | **R-CHOP-14**  **(*N*=303)**  ***n* (%)** |
| --- | --- | --- |
| Alive without progression | 184 (61%) | 182 (60%) |
| Alive after progression | 14 (5%) | 18 (6%) |
| Dead | 103 (34%) | 103 (34%) |
| Non-Hodgkin Lymphoma | 58 | 52 |
| Treatment related toxicity | 3 | 7 |
| Secondary Malignancy | 10 | 11 |
| Cardiac Death | 6 | 7 |
| Other | 26 | 22 |
| Missing | 0 | 4 |

**Table S4: Overall survival**

|  | **Univariable** | | **Multivariable** | |
| --- | --- | --- | --- | --- |
| **Variable** | **HR (95% CI)** | ***P*** | **HR (95% CI)** | ***P*** |
| R-CHOP-14 arm | 0.95 (0.73-1.25) | 0.74 | 1.11 (0.78-1.60) | 0.55 |
| Age (per year) | 1.07 (1.05-1.09) | <0.0001 | 1.05 (1.02-1.08) | <0.01 |
| Stage III/IV | 1.30 (0.97-1.76) | 0.08 | 1.25 (0.82-1.89) | 0.30 |
| PS >1 | 1.51 (1.08-2.13) | 0.02 | 1.01 (0.63-1.64) | 0.96 |
| LDH >ULN | 1.60 (1.17-2.18) | <0.01 | 1.37 (0.89-2.11) | 0.15 |
| Extranodal sites >1 | 1.33 (1.00-1.77) | 0.05 | 1.01 (0.68-1.50) | 0.96 |
| B2M ≥3mg/L | 2.15 (1.48-3.12) | <0.0001 | 1.54 (1.02-2.33) | 0.04 |
| Bulky disease | 1.08 (0.82-1.43) | 0.57 | 0.98 (0.67-1.43) | 0.92 |
| Male sex | 1.13 (0.86-1.48) | 0.39 | 1.03 (0.72-1.47) | 0.88 |
| Albumin ≤35g/L | 1.79 (1.35-2.36) | <0.0001 | 1.43 (0.97-2.12) | 0.07 |

**Table S5: Performance of prognostic scores**

| **Groups**  **(no. of factors)** | **Patients**  ***n* (%)** | **CPE** | **AIC** |
| --- | --- | --- | --- |
| **IPI** (age ≥60y, WHO PS ˃1, Stage III/IV, LDH ˃ULN*, extranodal sites ˃1) | | | |
| Low (0-1) | 92 (15) |  |  |
| Low-intermediate (2) | 165 (27) | 0.571 | 2486 |
| High-intermediate (3) | 202 (33) |  |  |
| High (4-5) | 145 (24) |  |  |
| **R-IPI** (age ≥60y, WHO PS ˃1, Stage III/IV, LDH ˃ULN, extranodal sites ˃1) | | | |
| Very good (0) | 0 (0) |  |  |
| Good (1-2) | 257 (43) | 0.558 | 2488 |
| Poor (3-5) | 347 (57) |  |  |
| **E-IPI** (age ≥70y, WHO PS ˃1, Stage III/IV, LDH ˃ULN, extranodal sites ˃1) | | | |
| Low (0-1) | 150 (25) |  |  |
| Low-intermediate (2) | 180 (30) | 0.546 | 2494 |
| High-intermediate (3) | 175 (29) |  |  |
| High (4-5) | 99 (16) |  |  |
| **ABE4** (age ≥70y, WHO PS ≥1, bulky disease) | | | |
| Low (0) | 114 (19) |  |  |
| Low-intermediate (1) | 215 (36) | 0.593 | 2479 |
| High-intermediate (2) | 218 (36) |  |  |
| High (3) | 57 (9) |  |  |

*ULN indicates upper limit of normal.

**Figure S1: Kaplan-Meier curves of PFS and OS according to COO subtypes**

**
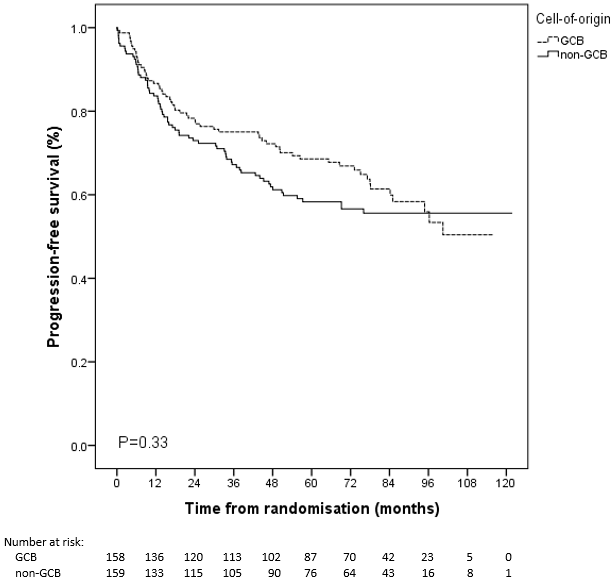

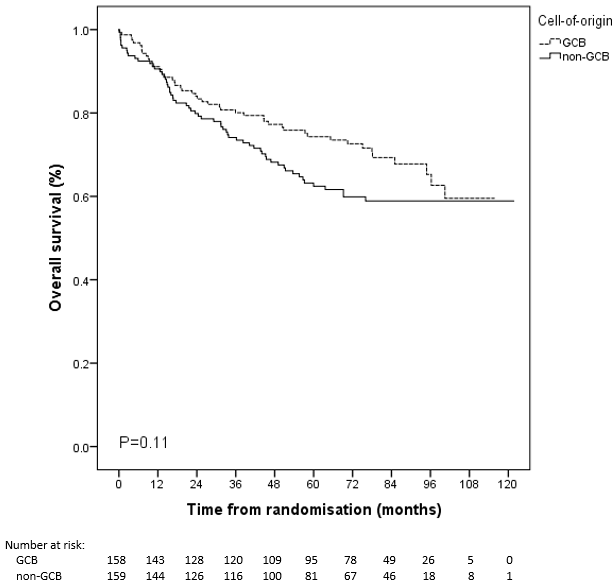
**

**Figure S2: Kaplan-Meier curves of OS according to different prognostic scores**

**
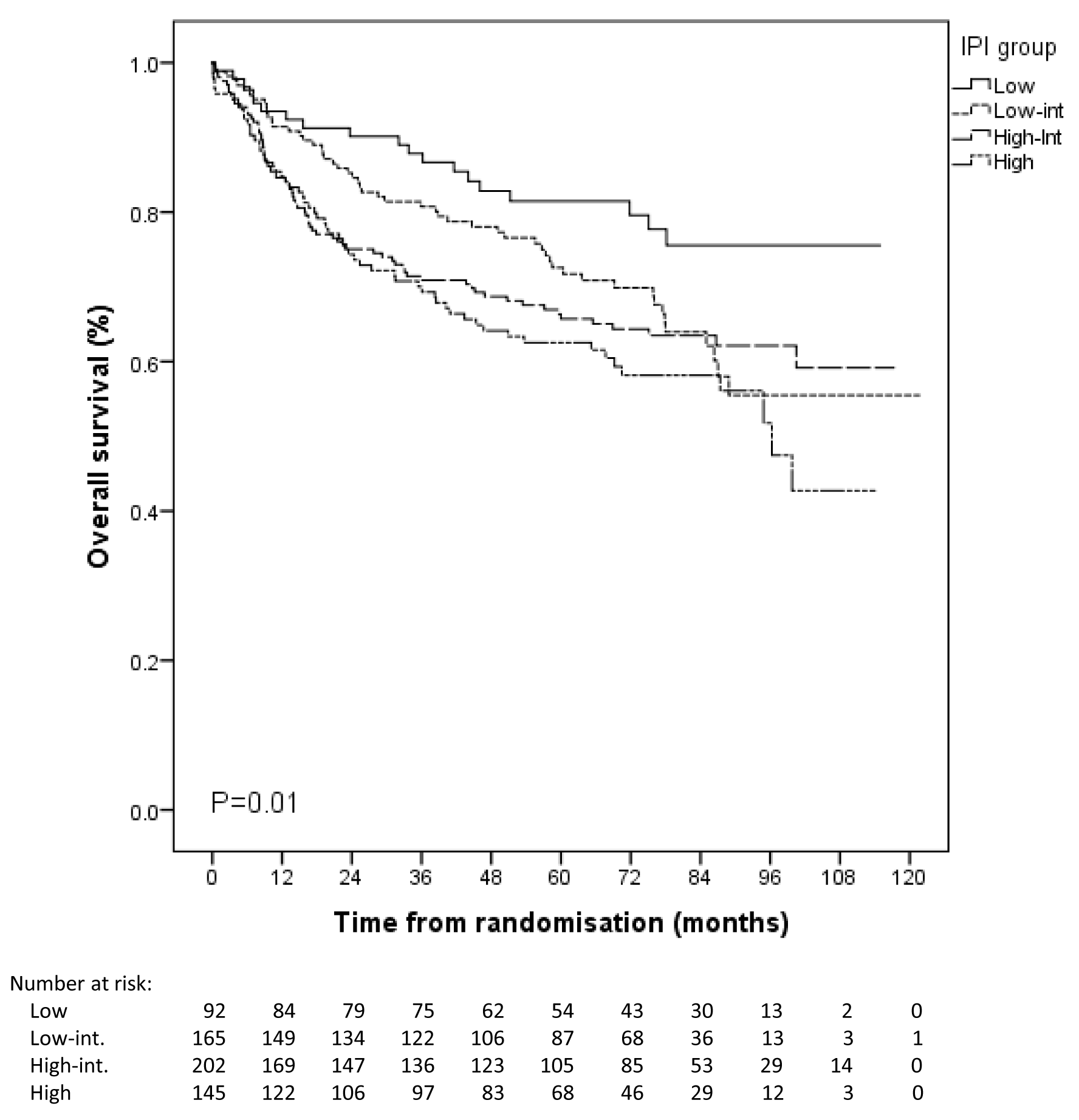

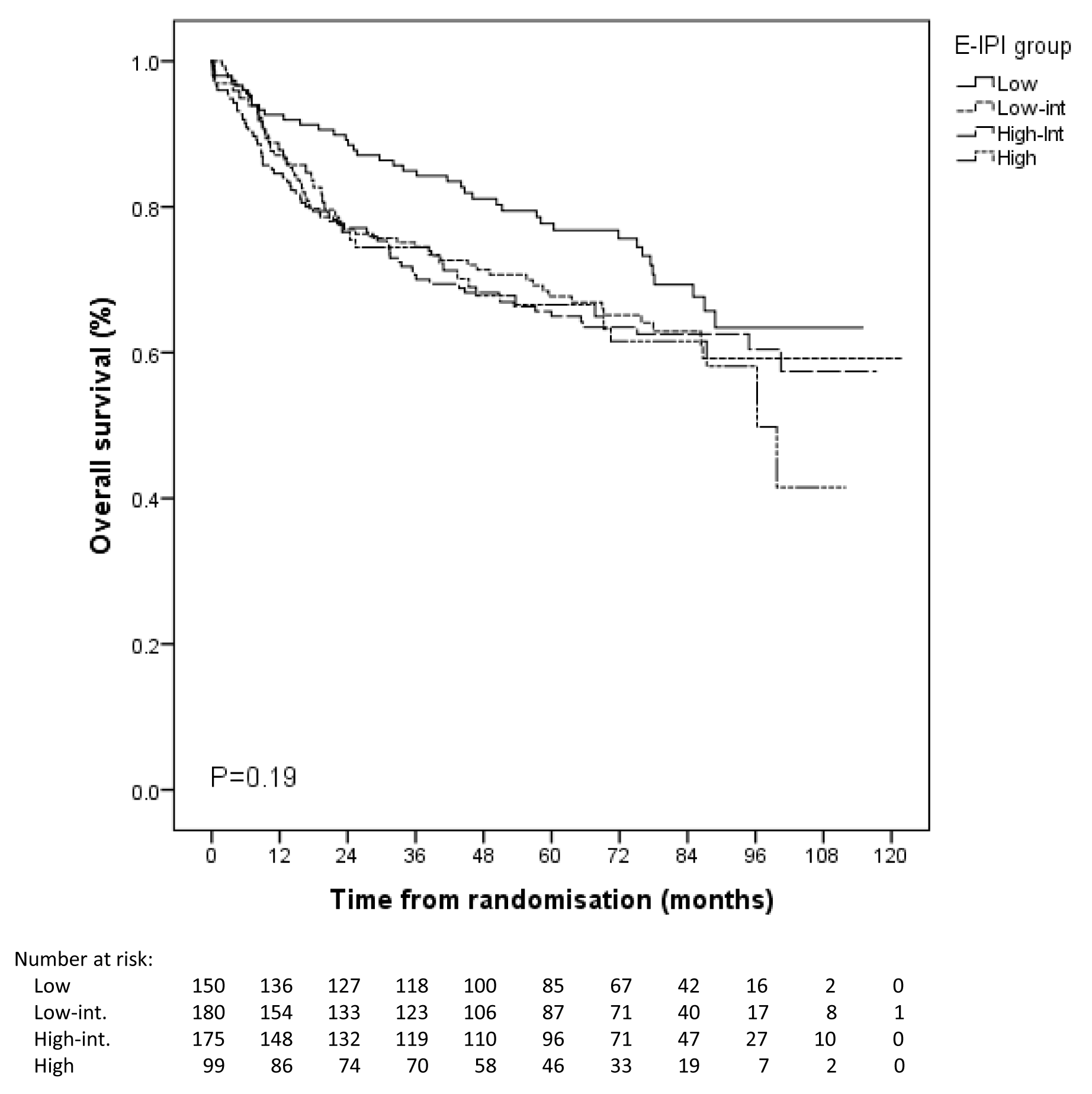
**

**
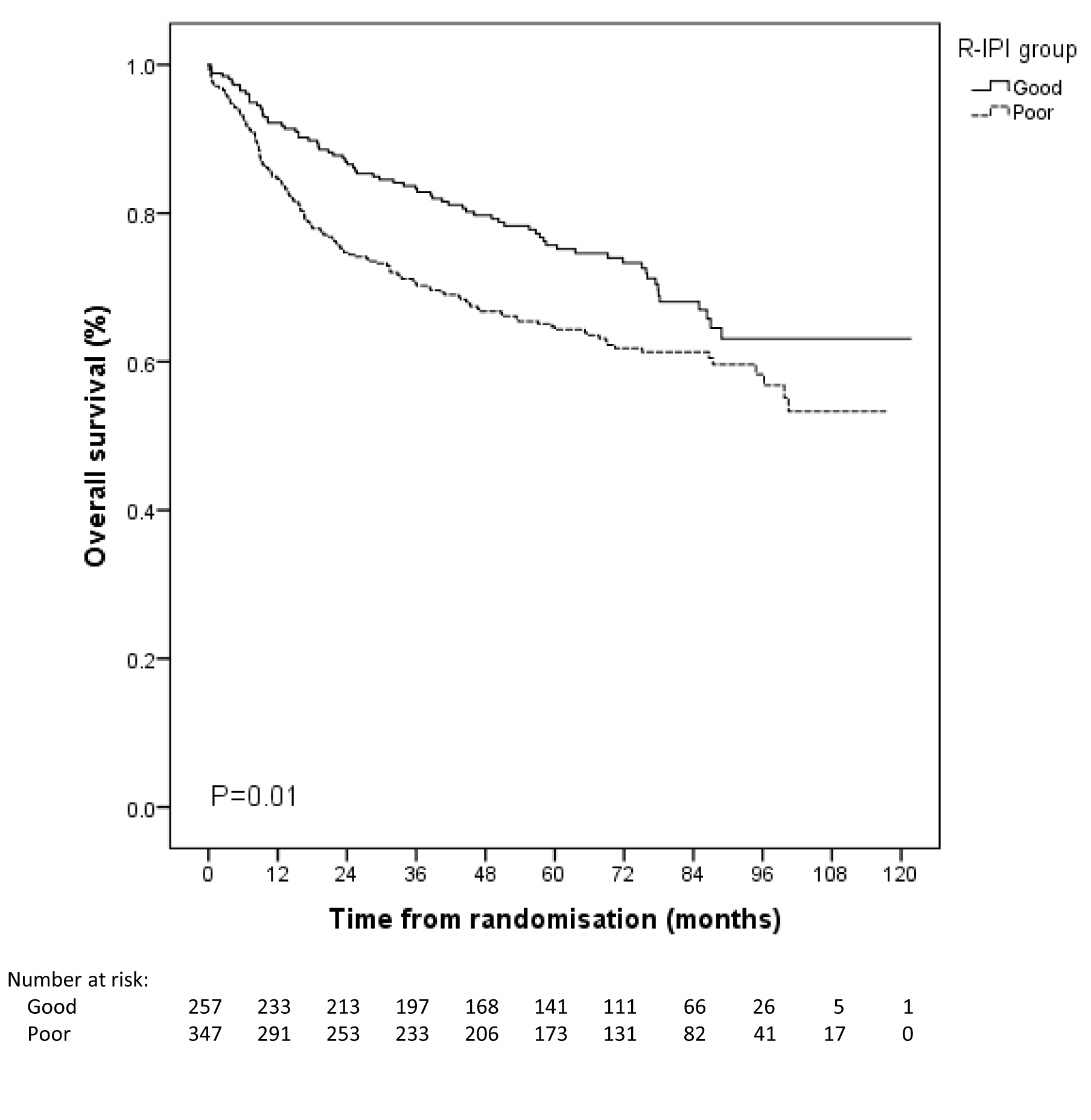

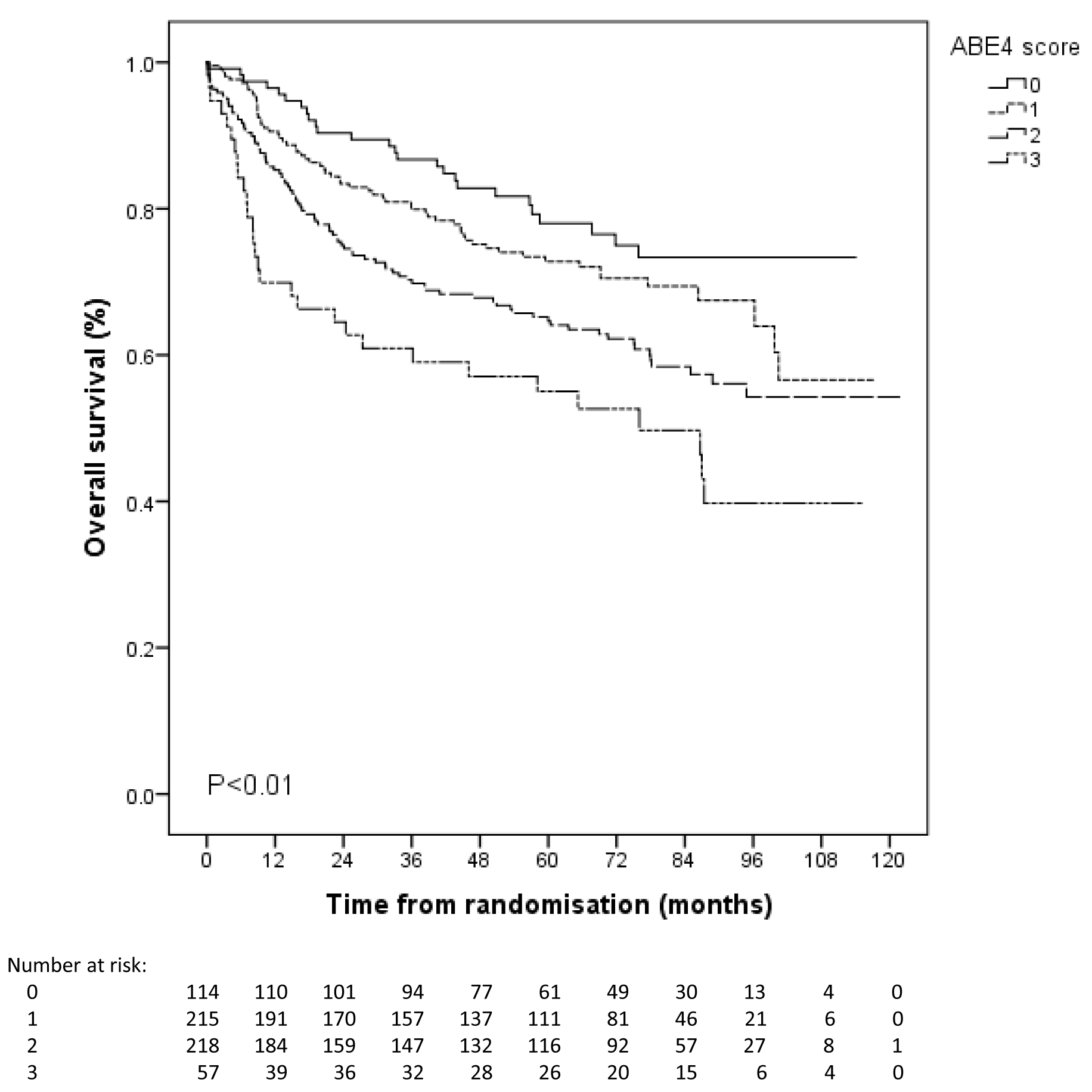
**

**Figure S3: Kaplan-Meier curves of PFS and OS according to consolidation radiotherapy (RT) in patients with PR or SD at the end of chemotherapy**

**
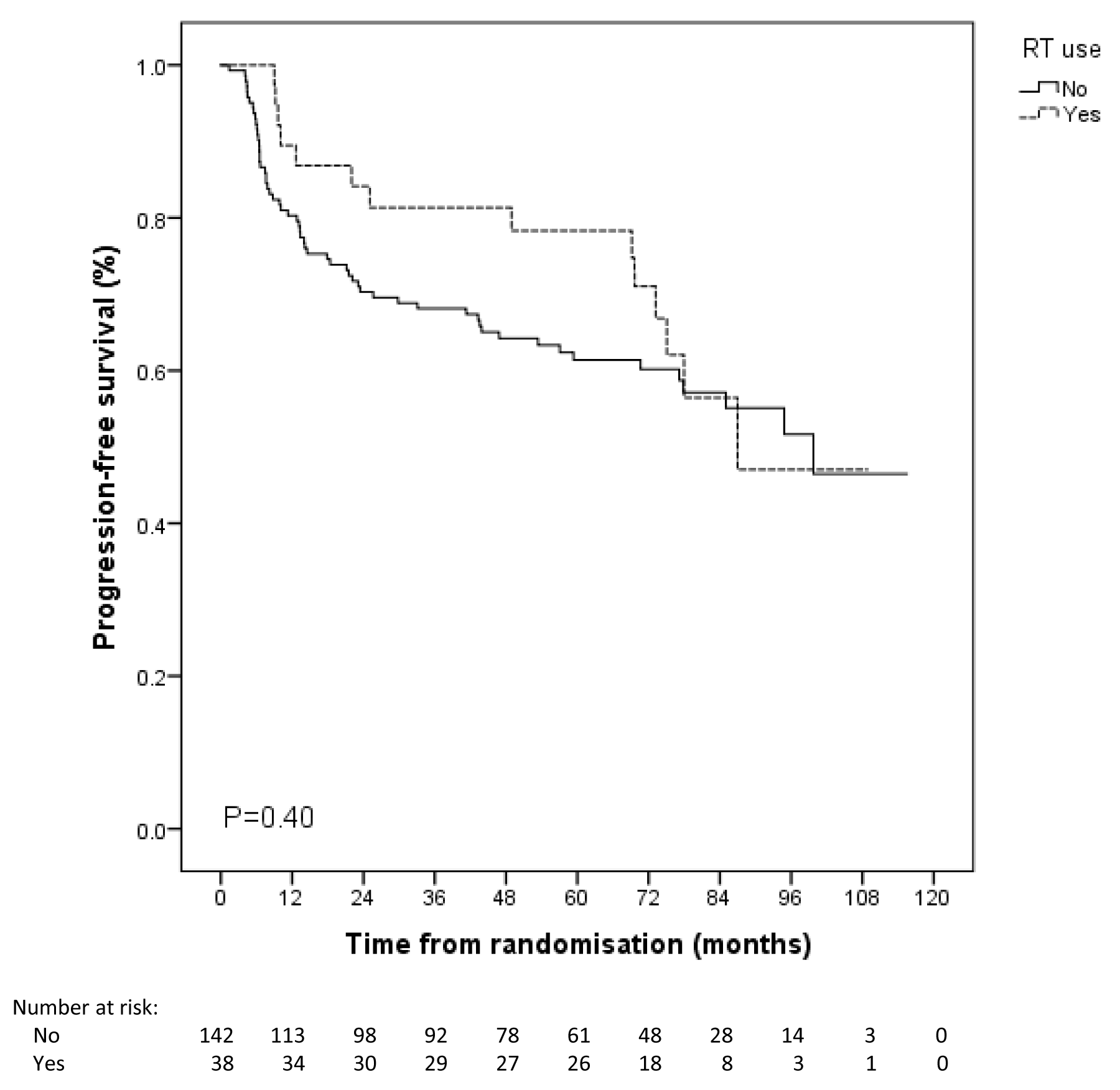

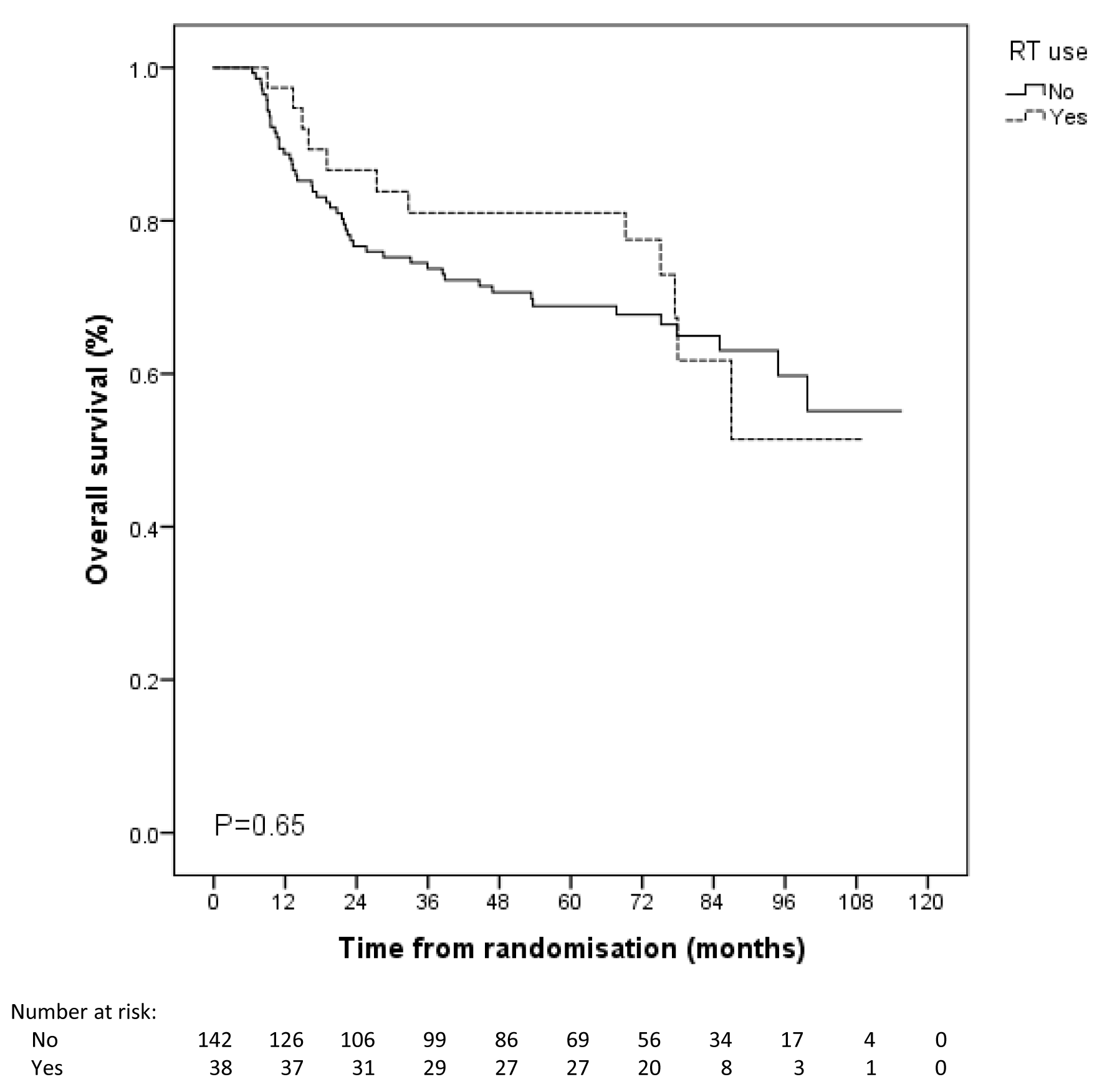
**
