# Supplementary material for: Outcome of elderly patients with diffuse large B-cell lymphoma treated with R-CHOP: results from the UK NCRI R-CHOP14v21 trial with combined analysis of molecular characteristics with the DSHNHL RICOVER-60 trial
Source: Ann Oncol. 2017 Apr 7;28(7):1540–6. doi: 10.1093/annonc/mdx128 (PMC5815562; doi:10.1093/annonc/mdx128)
Supplement: Supplementary Data [file mdx128_supp.zip › mdx128-suppl_data/Table S1.docx]

**Table S1: Clinical characteristics according to MYC-R and DHL**

| **Characteristics** | **non-MYC**  **(*N*=379)**  *n* (%) | **MYC-R**  **(*N*=42)**  *n* (%) | **non-DHL**  **(*N*=374)**  *n* (%) | **DHL**  **(*N*=23)**  *n* (%) |
| --- | --- | --- | --- | --- |
| Age (years)  60-69  ≥70 | 233 (61%)  146 (39%) | 25 (60%)  17 (40%) | 230 (61%)  144 (39%) | 14 (61%)  9 (39%) |
| Sex  Female  Male | 197 (52%)  182 (48%) | 18 (43%)  24 (57%) | 196 (52%)  178 (48%) | 11 (48%)  12 (52%) |
| Stage III/IV | 203 (54%) | 19 (45%) | 201 (54%) | 13 (57%) |
| WHO performance status >1 | 40 (11%) | 7 (17%) | 39 (10%) | 4 (17%) |
| Elevated LDH | 201 (53%) | 26 (62%) | 205 (55%) | 15 (65%) |
| >1 extranodal sites | 83 (22%) | 9 (21%) | 80 (21%) | 7 (30%) |
| IPI score  1  2  3  4  5 | 110 (29%)  93 (25%)  110 (29%)  57 (15%)  9 (2%) | 13 (31%)  10 (24%)  10 (24%)  6 (14%)  3 (7%) | 105 (28%)  95 (25%)  110 (29%)  54 (14%)  10 (3%) | 5 (22%)  5 (22%)  7 (30%)  4 (17%)  2 (9%) |
| Bulk | 118 (31%) | 19 (45%) | 121 (32%) | 11 (48%) |
| B symptoms | 120 (32%) | 17 (40%) | 118 (32%) | 9 (39%) |
