# Supplementary material for: Outcome of elderly patients with diffuse large B-cell lymphoma treated with R-CHOP: results from the UK NCRI R-CHOP14v21 trial with combined analysis of molecular characteristics with the DSHNHL RICOVER-60 trial
Source: Ann Oncol. 2017 Apr 7;28(7):1540–6. doi: 10.1093/annonc/mdx128 (PMC5815562; doi:10.1093/annonc/mdx128)
Supplement: Supplementary Data [file mdx128_supp.zip › mdx128-suppl_data/Table S2.docx]

**Table S2: Reasons for early termination of treatment**

| **Reason for early termination** | **R-CHOP-21**  **(*N*=301)**  ***n*** | **R-CHOP-14**  **(*N*=303)**  ***n*** |
| --- | --- | --- |
| Disease progression (PD)  -Death due to PD | 7  1 | 4  2 |
| Clinical decision | 8 | 1 |
| Patient refusal | 6 | 5 |
| Other medical condition  -Death due to other medical condition | 10  3 | 9  3 |
| Treatment-related toxicity  -Death related to treatment | 27  3 | 19  7 |
| Death, other cause or unknown  Diagnosis changed  Other | 2  3  7 | 1  4  2 |
| Not known/missing | 1 | 1 |
