# Supplementary material for: Outcome of elderly patients with diffuse large B-cell lymphoma treated with R-CHOP: results from the UK NCRI R-CHOP14v21 trial with combined analysis of molecular characteristics with the DSHNHL RICOVER-60 trial
Source: Ann Oncol. 2017 Apr 7;28(7):1540–6. doi: 10.1093/annonc/mdx128 (PMC5815562; doi:10.1093/annonc/mdx128)
Supplement: Supplementary Data [file mdx128_supp.zip › mdx128-suppl_data/Table S3.docx]

**Table S3: Survival status and cause of death**

| **Status and cause of death** | **R-CHOP-21**  **(*N*=301)**  ***n* (%)** | **R-CHOP-14**  **(*N*=303)**  ***n* (%)** |
| --- | --- | --- |
| Alive without progression | 184 (61%) | 182 (60%) |
| Alive after progression | 14 (5%) | 18 (6%) |
| Dead | 103 (34%) | 103 (34%) |
| Non-Hodgkin Lymphoma | 58 | 52 |
| Treatment related toxicity | 3 | 7 |
| Secondary Malignancy | 10 | 11 |
| Cardiac Death | 6 | 7 |
| Other | 26 | 22 |
| Missing | 0 | 4 |
