# Supplementary material for: Outcome of elderly patients with diffuse large B-cell lymphoma treated with R-CHOP: results from the UK NCRI R-CHOP14v21 trial with combined analysis of molecular characteristics with the DSHNHL RICOVER-60 trial
Source: Ann Oncol. 2017 Apr 7;28(7):1540–6. doi: 10.1093/annonc/mdx128 (PMC5815562; doi:10.1093/annonc/mdx128)
Supplement: Supplementary Data [file mdx128_supp.zip › mdx128-suppl_data/Table S4.docx]

**Table S4: Overall survival**

|  | **Univariable** | | **Multivariable** | |
| --- | --- | --- | --- | --- |
| **Variable** | **HR (95% CI)** | ***P*** | **HR (95% CI)** | ***P*** |
| R-CHOP-14 arm | 0.95 (0.73-1.25) | 0.74 | 1.11 (0.78-1.60) | 0.55 |
| Age (per year) | 1.07 (1.05-1.09) | <0.0001 | 1.05 (1.02-1.08) | <0.01 |
| Stage III/IV | 1.30 (0.97-1.76) | 0.08 | 1.25 (0.82-1.89) | 0.30 |
| PS >1 | 1.51 (1.08-2.13) | 0.02 | 1.01 (0.63-1.64) | 0.96 |
| LDH >ULN | 1.60 (1.17-2.18) | <0.01 | 1.37 (0.89-2.11) | 0.15 |
| Extranodal sites >1 | 1.33 (1.00-1.77) | 0.05 | 1.01 (0.68-1.50) | 0.96 |
| B2M ≥3mg/L | 2.15 (1.48-3.12) | <0.0001 | 1.54 (1.02-2.33) | 0.04 |
| Bulky disease | 1.08 (0.82-1.43) | 0.57 | 0.98 (0.67-1.43) | 0.92 |
| Male sex | 1.13 (0.86-1.48) | 0.39 | 1.03 (0.72-1.47) | 0.88 |
| Albumin ≤35g/L | 1.79 (1.35-2.36) | <0.0001 | 1.43 (0.97-2.12) | 0.07 |
