# Supplementary material for: Outcome of elderly patients with diffuse large B-cell lymphoma treated with R-CHOP: results from the UK NCRI R-CHOP14v21 trial with combined analysis of molecular characteristics with the DSHNHL RICOVER-60 trial
Source: Ann Oncol. 2017 Apr 7;28(7):1540–6. doi: 10.1093/annonc/mdx128 (PMC5815562; doi:10.1093/annonc/mdx128)
Supplement: Supplementary Data [file mdx128_supp.zip › mdx128-suppl_data/Table S5.docx]

**Table S5: Performance of prognostic scores**

| **Groups**  **(no. of factors)** | **Patients**  ***n* (%)** | **CPE** | **AIC** |
| --- | --- | --- | --- |
| **IPI** (age ≥60y, WHO PS ˃1, Stage III/IV, LDH ˃ULN*, extranodal sites ˃1) | | | |
| Low (0-1) | 92 (15) |  |  |
| Low-intermediate (2) | 165 (27) | 0.571 | 2486 |
| High-intermediate (3) | 202 (33) |  |  |
| High (4-5) | 145 (24) |  |  |
| **R-IPI** (age ≥60y, WHO PS ˃1, Stage III/IV, LDH ˃ULN, extranodal sites ˃1) | | | |
| Very good (0) | 0 (0) |  |  |
| Good (1-2) | 257 (43) | 0.558 | 2488 |
| Poor (3-5) | 347 (57) |  |  |
| **E-IPI** (age ≥70y, WHO PS ˃1, Stage III/IV, LDH ˃ULN, extranodal sites ˃1) | | | |
| Low (0-1) | 150 (25) |  |  |
| Low-intermediate (2) | 180 (30) | 0.546 | 2494 |
| High-intermediate (3) | 175 (29) |  |  |
| High (4-5) | 99 (16) |  |  |
| **ABE4** (age ≥70y, WHO PS ≥1, bulky disease) | | | |
| Low (0) | 114 (19) |  |  |
| Low-intermediate (1) | 215 (36) | 0.593 | 2479 |
| High-intermediate (2) | 218 (36) |  |  |
| High (3) | 57 (9) |  |  |

*ULN indicates upper limit of normal.
