# Supplementary figures and images for: Outcome of elderly patients with diffuse large B-cell lymphoma treated with R-CHOP: results from the UK NCRI R-CHOP14v21 trial with combined analysis of molecular characteristics with the DSHNHL RICOVER-60 trial
Source: Ann Oncol. 2017 Apr 7;28(7):1540–6. doi: 10.1093/annonc/mdx128 (PMC5815562; doi:10.1093/annonc/mdx128)

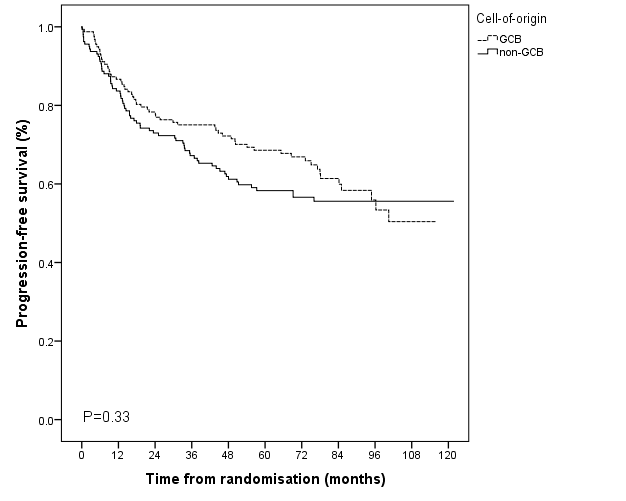

Supplement: Supplementary Data [file mdx128_supp.zip › mdx128-suppl_data/Figure S1_1.tif]

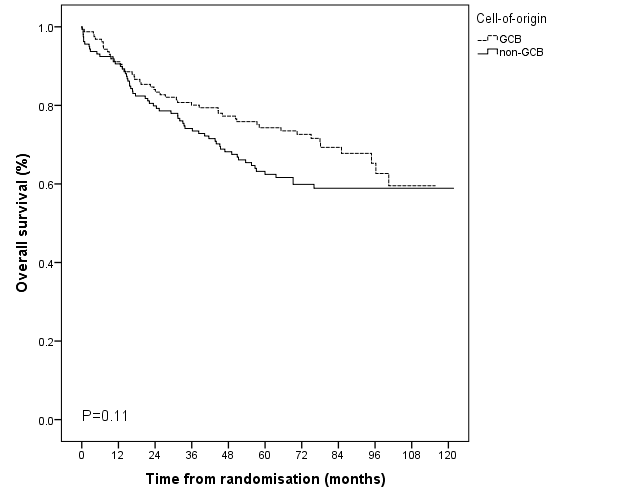

Supplement: Supplementary Data [file mdx128_supp.zip › mdx128-suppl_data/Figure S1_2.tif]

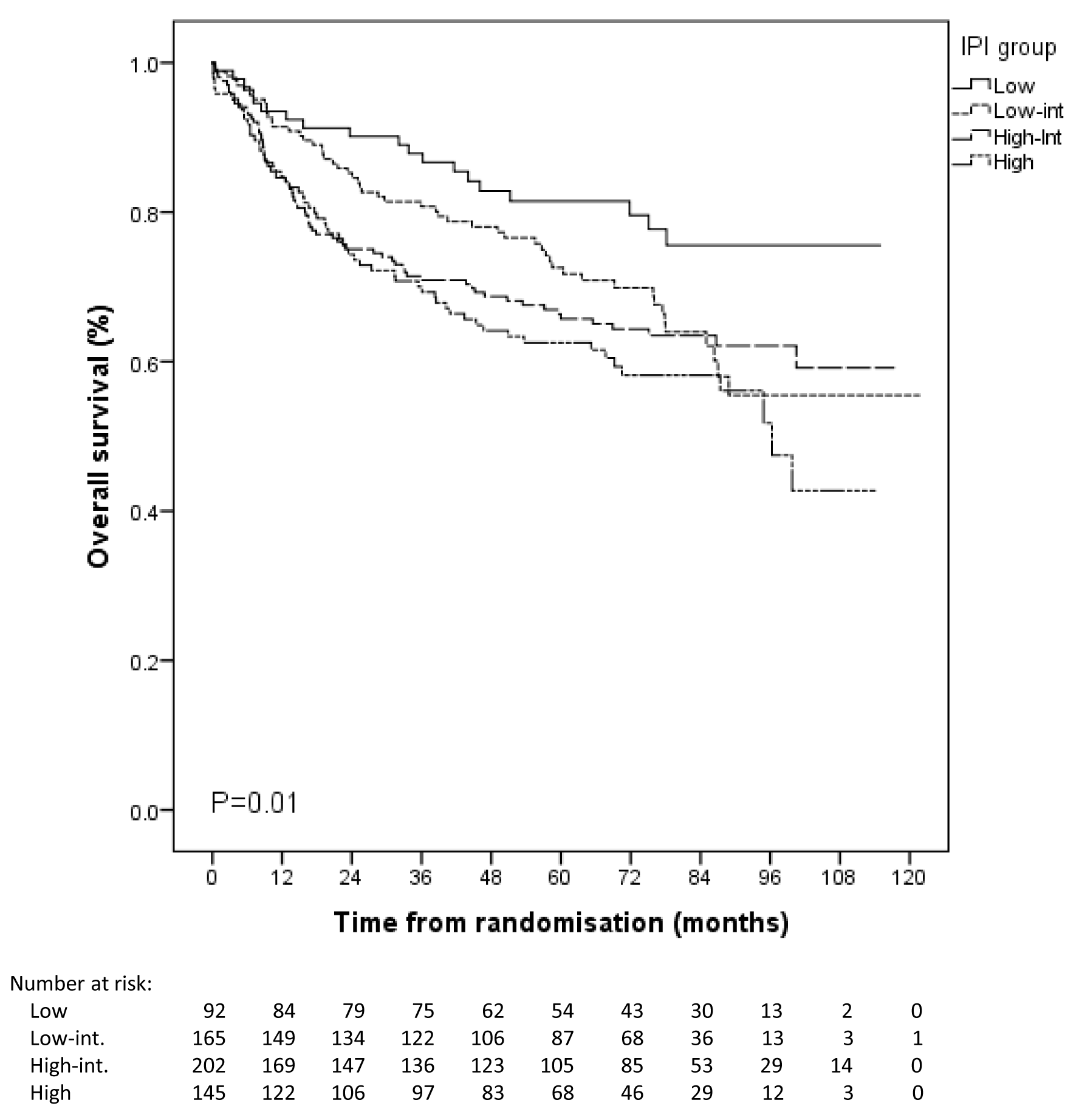

Supplement: Supplementary Data [file mdx128_supp.zip › mdx128-suppl_data/Figure S2_1.tif]

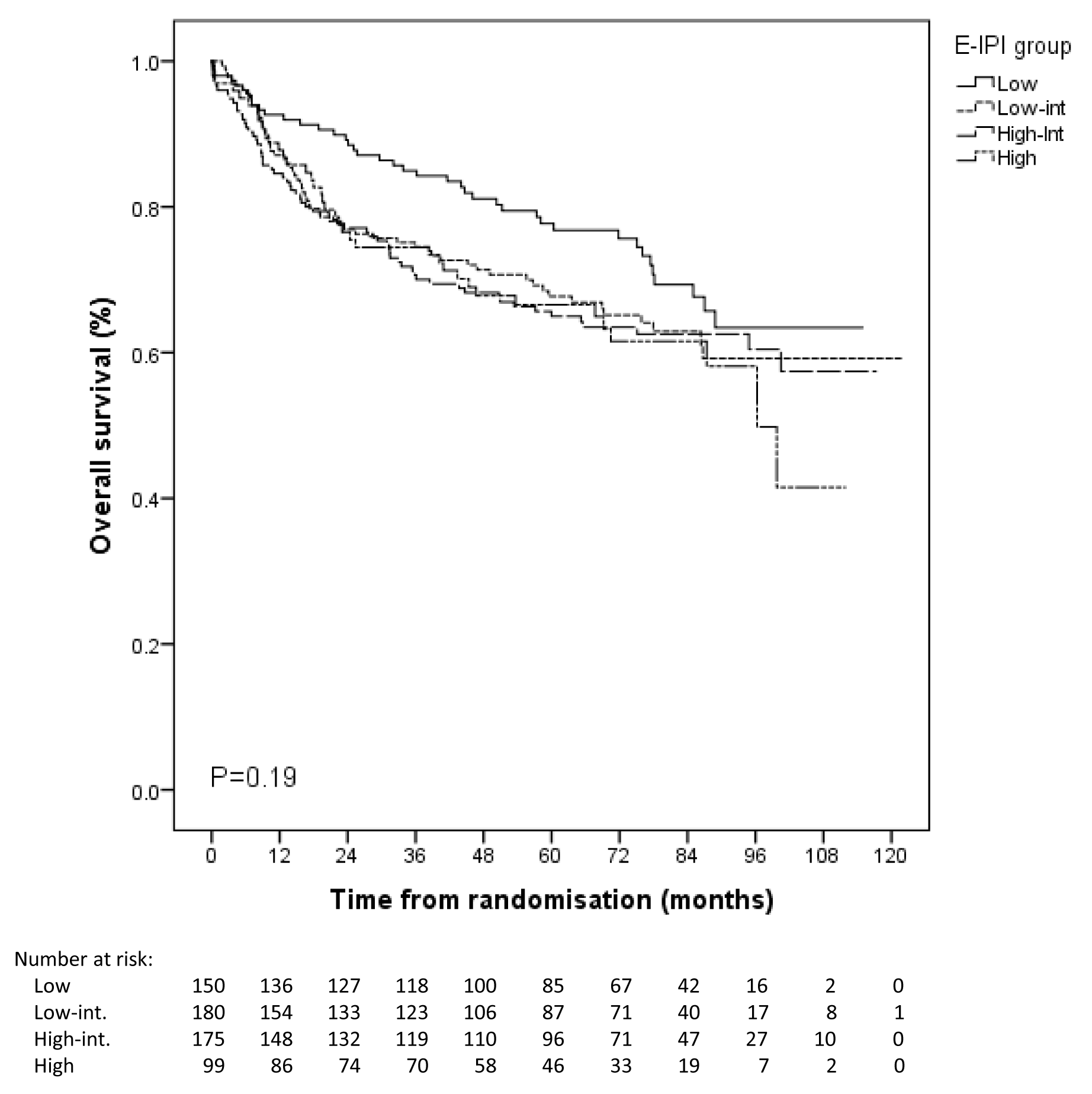

Supplement: Supplementary Data [file mdx128_supp.zip › mdx128-suppl_data/Figure S2_2.tif]

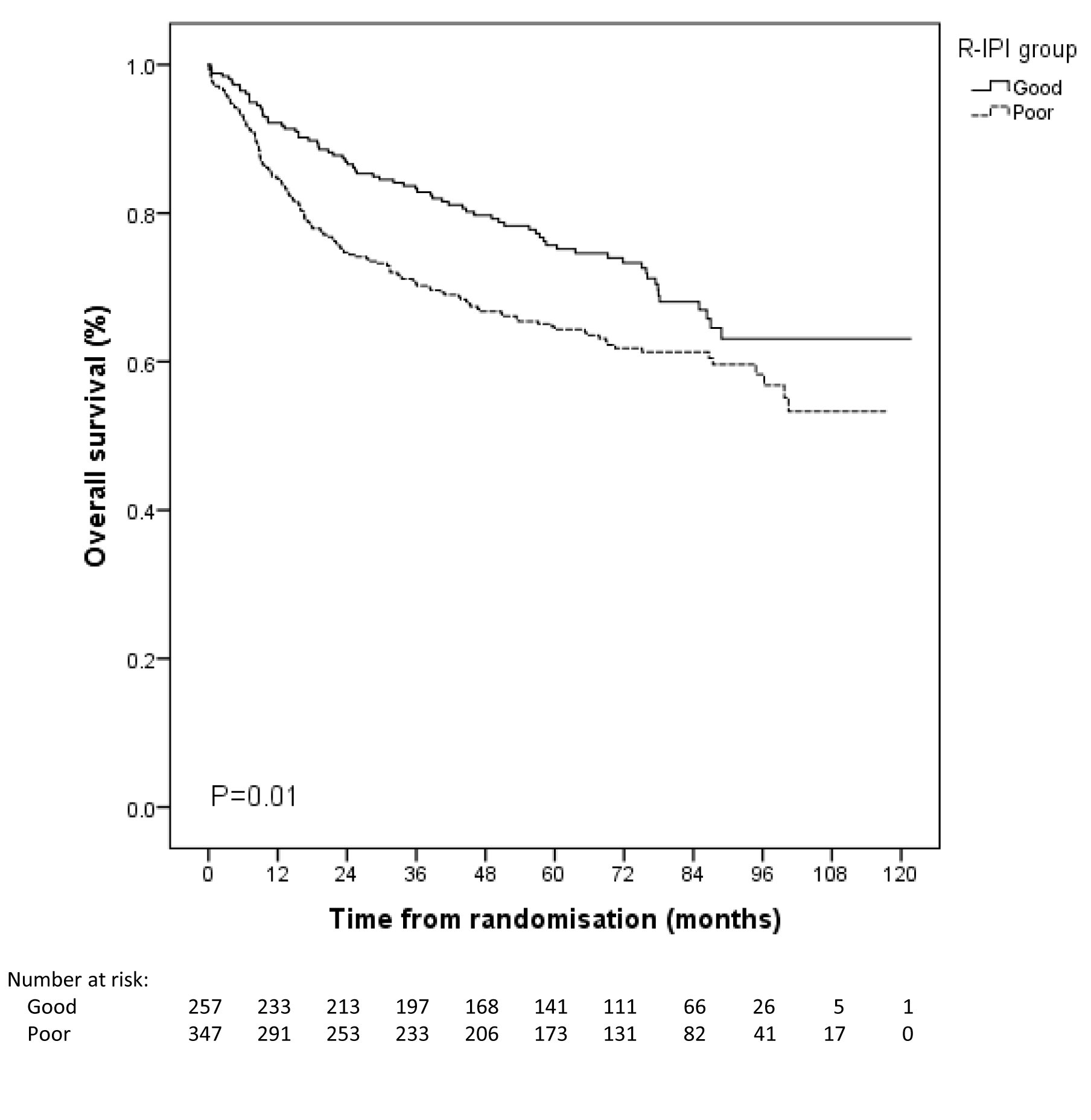

Supplement: Supplementary Data [file mdx128_supp.zip › mdx128-suppl_data/Figure S2_3.tif]

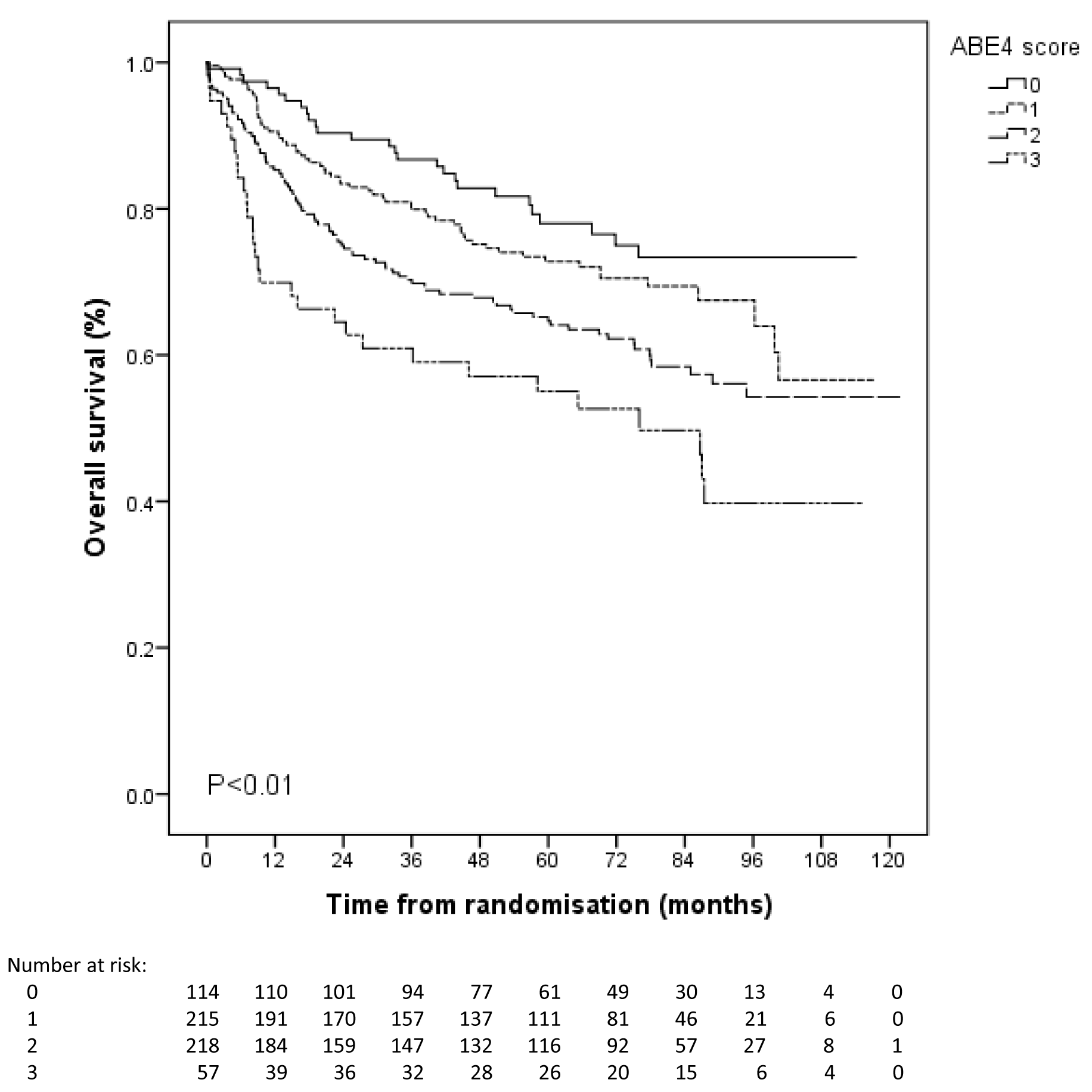

Supplement: Supplementary Data [file mdx128_supp.zip › mdx128-suppl_data/Figure S2_4.tif]

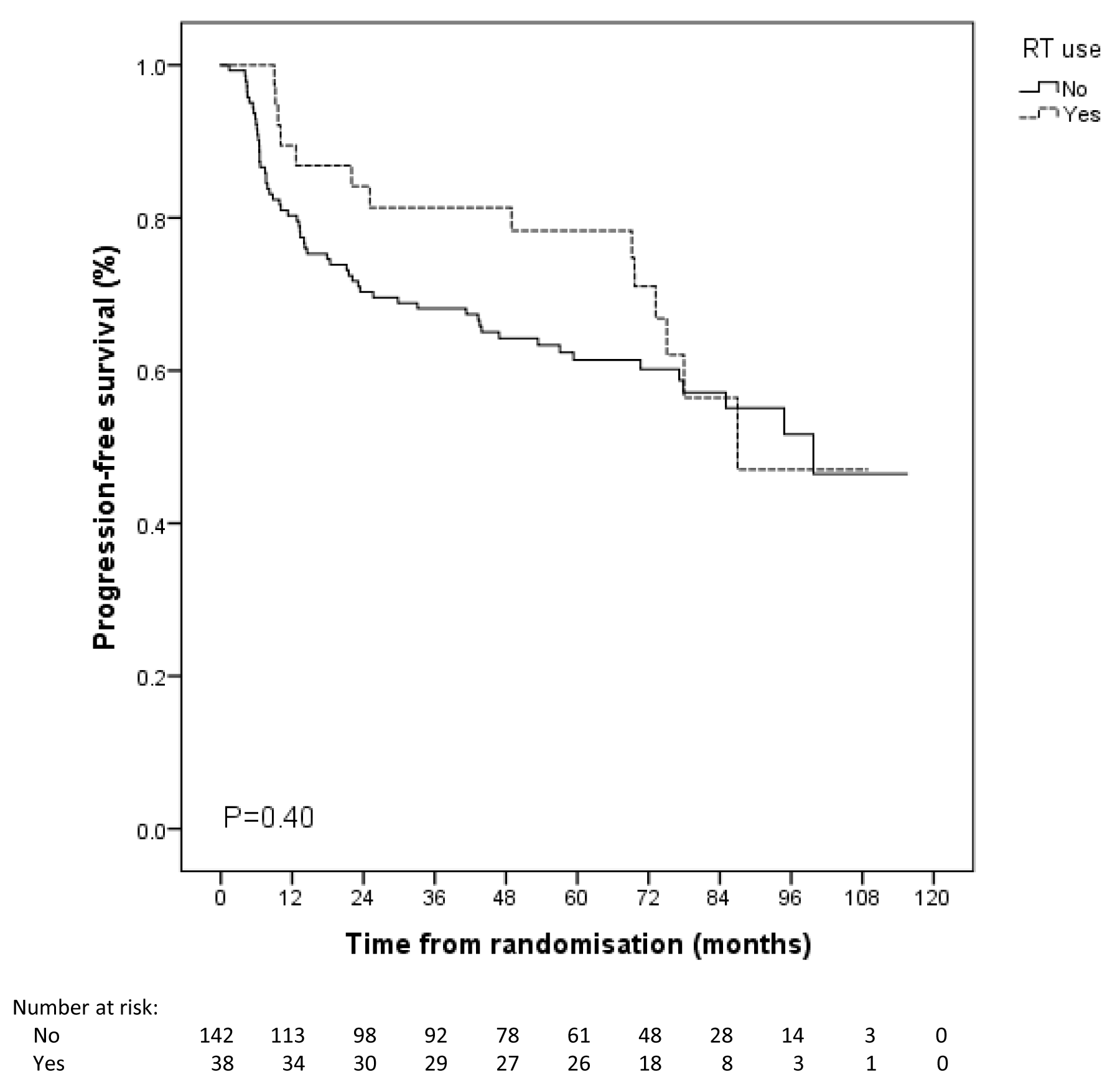

Supplement: Supplementary Data [file mdx128_supp.zip › mdx128-suppl_data/Figure S3_1.tif]

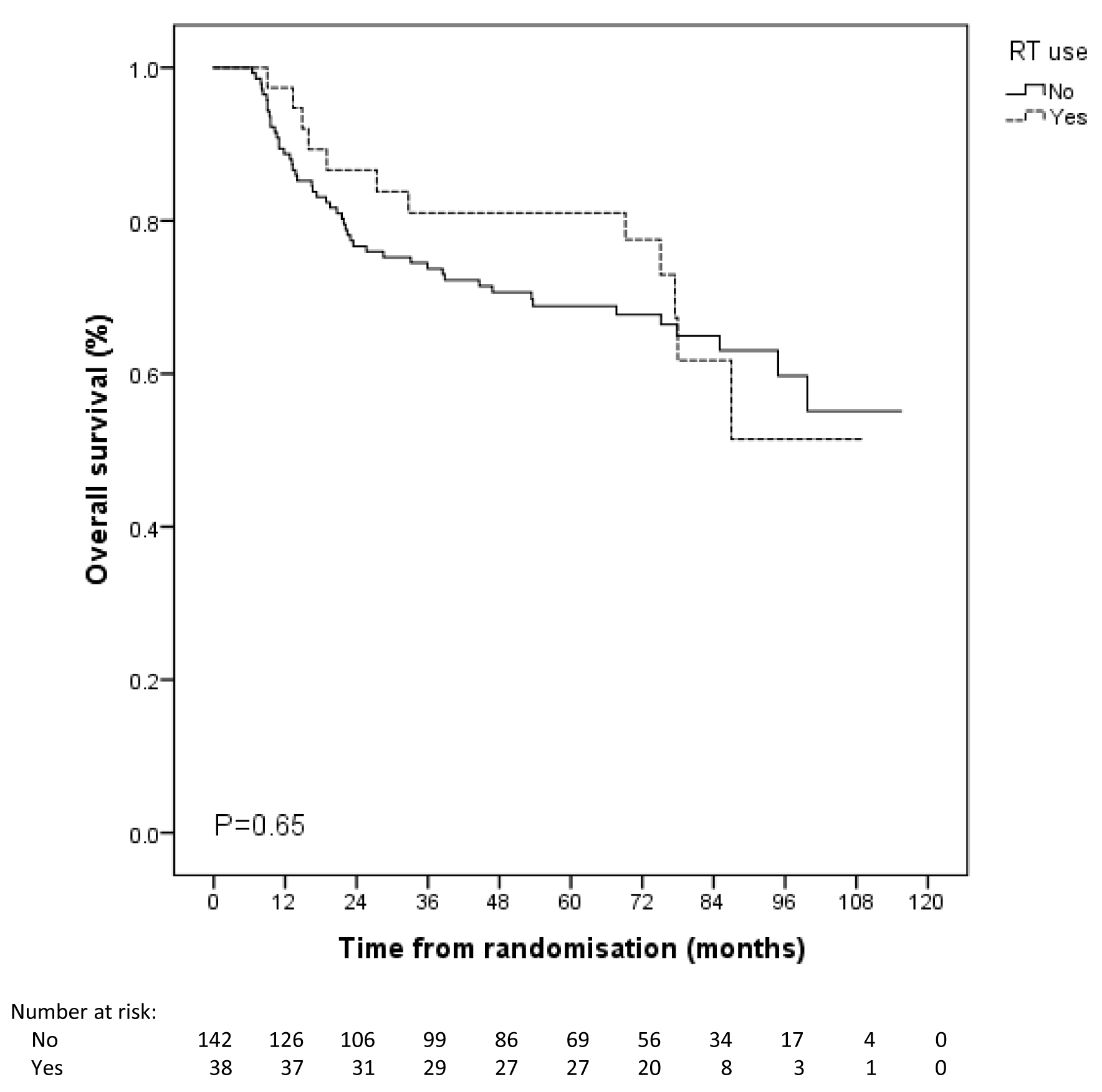

Supplement: Supplementary Data [file mdx128_supp.zip › mdx128-suppl_data/Figure S3_2.tif]
